# Do Ask, Do Tell: High Levels of Acceptability by Patients of Routine Collection of Sexual Orientation and Gender Identity Data in Four Diverse American Community Health Centers

**DOI:** 10.1371/journal.pone.0107104

**Published:** 2014-09-08

**Authors:** Sean Cahill, Robbie Singal, Chris Grasso, Dana King, Kenneth Mayer, Kellan Baker, Harvey Makadon

**Affiliations:** 1 The Fenway Institute, Northeastern University Department of Political Science, Boston, MA, United States of America/New York University Wagner School, New York, NY, United States of America; 2 The Fenway Institute, Boston, MA, United States of America; 3 The Fenway Institute/Beth Israel Deaconess Medical Center/Harvard Medical School, Boston, MA, United States of America; 4 Center for American Progress, Washington, DC, United States of America; 5 The Fenway Institute/Harvard Medical School, Boston, MA, United States of America; The University of New South Wales, Australia

## Abstract

**Background:**

The Institute of Medicine and The Joint Commission have recommended asking sexual orientation and gender identity (SOGI) questions in clinical settings and including such data in Electronic Health Records (EHRs). This is increasingly viewed as a critical step toward systematically documenting and addressing health disparities affecting lesbian, gay, bisexual, and transgender (LGBT) people. The U.S. government is currently considering whether to include SOGI data collection in the Stage 3 guidelines for the incentive program promoting meaningful use of EHR. However, some have questioned whether acceptable standard measures to collect SOGI data in clinical settings exist.

**Methods:**

In order to better understand how a diverse group of patients would respond if SOGI questions were asked in primary care settings, 301 randomly selected patients receiving primary care at four health centers across the U.S. were asked SOGI questions and then asked follow-up questions. This sample was mainly heterosexual, racially diverse, and geographically and regionally broad.

**Results:**

There was a strong consensus among patients surveyed about the importance of asking SOGI questions. Most of the LGBT respondents thought that the questions presented on the survey allowed them to accurately document their SOGI. Most respondents—heterosexual and LGBT—answered the questions, and said that they would answer such questions in the future. While there were some age-related differences, respondents of all ages overwhelmingly expressed support for asking SOGI questions and understood the importance of providers' knowing their patients' SOGI.

**Conclusions:**

Given current deliberations within national health care regulatory bodies and the government's increased attention to LGBT health disparities, the finding that patients can and will answer SOGI questions has important implications for public policy. This study provides evidence that integrating SOGI data collection into the meaningful use requirements is both acceptable to diverse samples of patients, including heterosexuals, and feasible.

## Introduction

A 2011 Institute of Medicine report highlighted LGBT health disparities and encouraged routine collection of data on sexual orientation and gender identity (SOGI) in health care settings to better understand and address LGBT health.

The shift from paper to Electronic Health Records (EHR), initiated years ago and accelerated by funding from the American Recovery and Reinvestment Act of 2009 and the Patient Protection and Affordable Care Act of 2010, is a critical structural change in health care delivery that should help improve patient outcomes, reduce costs, and address health disparities [1]. The 2011 Institute of Medicine report on LGBT health recommended SOGI data collection in EHRs as part of the meaningful use objectives for the EHR Incentive Program run by the Office of the National Coordinator for Health Information Technology (ONC) and the Centers for Medicare and Medicaid Services (CMS). The report recommended that questions be standardized to allow for the comparison and pooling of data to analyze the specific health needs of LGBT people [2]. Healthy People 2020 calls on health care providers to “appropriately inquir[e] about and be…supportive of a patient's sexual orientation to enhance the patient-provider interaction and regular use of care.”[3] Gathering LGBT data in clinical settings is consistent with efforts of the U.S. Department of Health and Human Services to gather health data on LGBT populations as authorized under Section 4302 of the ACA [1]. Further, The Joint Commission's 2011 LGBT field guide encourages the collection of patient SOGI data [4]. Gathering SOGI data in clinical settings via EHR systems could help clinicians, researchers, and policymakers better understand LGBT health, including disparities in insurance coverage, access to care, diagnosis, and treatment. Storing SOGI information in the EHR should promote seamless communication among staff within health care organizations. Further, these data, coupled with race/ethnicity data, should also allow for better understandings of racial and ethnic disparities within LGBT populations.

One of the major reasons for the desirability of routinely collecting data about patient sexual orientation and gender identity is that there is a growing body of research that has documented health disparities affecting LGBT people [2]. These include:

- Gay and bisexual men experience high rates of mental and behavioral health issues, including depression and suicidal ideation [5].
- Lesbians and bisexual women experience cervical cancer at the same rate as heterosexual women, but are four to ten times less likely to get routine Pap tests to screen for cervical cancer [6], [7].
- Bisexual men and women may experience poorer health than homosexual and heterosexual respondents [8], as well as higher rates of mental health issues and smoking [9].
- Transgender people, particularly transgender women of color, are disproportionately likely to be victims of hate violence [10]. Transgender women and men are less likely to have access to preventive screenings that can detect diseases such as cancer early [11], and are more likely to attempt suicide [12].

LGBT people experience health disparities for many reasons. Minority stress related to social prejudice and attempts to conceal ones' sexual orientation or gender identity, as well as internalized homophobia, can correlate with mental health burden and substance use [13]. Gay and bisexual men and transgender women are at greater risk for HIV and other STIs because anal sex without a condom is a more efficient mode of transmitting STIs than vaginal sex [14]. Transgender women and men may be at elevated risk for cardiovascular disease related to exogenous hormone use [15].

Another factor in LGBT health disparities is discriminatory treatment in health care settings. Surveys of both patients [16] and providers [17] indicate that LGBT people experience prejudicial treatment in clinical settings and that some providers exhibit anti-LGBT bias. As a result, many LGBT people report culturally incompetent care, or avoid visiting health care facilities for fear of receiving substandard care [16]. The dearth of LGBT-inclusive cultural competency and clinical training for providers contributes to their widespread failure to discuss SOGI with their patients, perpetuating invisibility of LGBT patients in clinical settings. SOGI data collection is a key component of enhancing the ability of patients and providers to engage in meaningful dialogue in the exam room and to promote the provision of high-quality care for LGBT people [18]. Patient-provider discussions about SOGI can facilitate a more accurate assessment of patient self-reported health and risk behaviors [19].

It is important to study the most effective ways to gather SOGI information in clinical settings in order to advance SOGI data collection efforts that are useful from a staff and provider perspective as well as acceptable from a patient perspective [20].

While SOGI questions are currently asked in a variety of settings, such as demographic surveys, there is a need to specifically validate measures for use in EHRs and in clinical settings. The aim of this study, which surveyed diverse patient groups at 4 community health centers (CHCs) to assess the acceptability and feasibility of asking SOGI questions, is to evaluate a set of standardized SOGI questions that can be incorporated into EHRs at CHCs and potentially other health care organizations. A set of validated, standardized SOGI questions could allow for pooling of data in order to analyze the health needs of LGBT populations, evaluate the quality of care LGBT people receive, and better understand and address LGBT health disparities.

The current study was initiated as part of the Community Health Applied Research Network (CHARN), a group of community health centers funded by the Health Resources and Services Administration (HRSA) in 2010 to build capacity to conduct meaningful and rigorous multi-site Patient Centered Outcomes Research (PCOR) that should result in better patient care at federally-supported community health clinics with underserved patient populations. CHARN is comprised of seventeen community health centers in nine states that served 519,636 individual patients in 2010.

Participating sites in this study included Beaufort Jasper Hampton Comprehensive Health Services (Beaufort) in rural South Carolina; Chase Brexton Health Center (Chase Brexton) in Baltimore and Columbia, Maryland; Howard Brown Health Center in Chicago; and Fenway Health in Boston. Beaufort serves a predominantly heterosexual population. Fenway Health, Chase Brexton, and Howard Brown serve populations that include heterosexual and non-transgender patients, but have also developed expertise in the care of LGBT patients. All four health centers serve racially and ethnically diverse populations. The Fenway CHARN investigators developed the study proposal with input from the three CHARN clinical affiliate sites and the Center for American Progress, a non-profit think tank based in Washington, DC, focused on the implementation of progressive change, which also contributed financial support for the study.

## Methods

The specific aim of the study was to survey CHC patients to assess the acceptability, feasibility, and patient preferences on asking SOGI questions to complete their EHR registration. The study addressed the following questions:

1. What are the acceptable ways to ask patients about sexual orientation and gender identity and document their responses in EHR?
2. How do patient survey responses differ based on sexual orientation, gender identity, and other demographic variables?

### Participants

The findings from this study are based on survey responses from 301 patients at four CHARN-affiliated CHCs. By targeting CHCs with diverse patient populations, the goal was to enroll patients who are transgender (regardless of sexual orientation), LGB, and heterosexual to gather information on appropriate ways to ask SOGI questions. The inclusion criteria included patients at each participating CHC who were 18 year of age or older and able to read and comprehend English.

### Human subjects protection

The Fenway Institute functioned as the lead site for this study. Beaufort and Chase Brexton, both Fenway-affiliated CHARN sites, used the Fenway Institutional Review Board (IRB) for CHARN-related study projects. The Howard Brown Health Center IRB approved the study for that site.

### Instrument

This one-time, 5-minute survey asked respondents to answer a question about sexual orientation developed at the Fenway Institute, and to answer a two-step gender identity and birth sex question that has been endorsed by leading transgender researchers in the U.S. [21] and globally. [22] The sexual orientation question was already in use at Fenway but not at Beaufort and Chase Brexton. At Howard Brown, patients are encouraged to report their sexual orientation and their gender identity for inclusion in their EHR. All of the questions tested in the current study had not been tested among a diverse population at CHCs.

After extensive pilot testing, Fenway Health added the following question about sexual orientation to its patient registration form and EHR in 2011. [23] In the current study, we included this sexual orientation question in the survey administered at the four participating CHCs.

*Do you think of yourself as:*

□ *Lesbian, gay or homosexual*
□ *Straight or heterosexual*
□ *Bisexual*
□ *Something else, please describe________________________*
□ *Don't know*

In the current study, we also asked:

*What is your current gender identity?*

*(Check all that apply)*

□ *Male*
□ *Female*
□ *Female-to-Male (FTM)/Transgender Male/Trans Man*
□ *Male-to-Female (MTF)/Transgender Female/Trans Woman*
□ *Genderqueer, neither exclusively male nor female*
□ *Additional Gender Category/(or Other), please specify _____________*
□ *Decline to Answer, please explain why _____________*

*What sex were you assigned at birth on your original birth certificate?*

*(Check one)*

□ *Male*
□ *Female*
□ *Decline to Answer, please explain why _____________*

Respondents were also asked a number of clarifying questions about these sexual orientation and gender identity questions in order to gauge comprehension, acceptability, and whether they thought the question allowed them to accurately document their sexual orientation, gender identity, and, ultimately, their health needs in an electronic health record system. In addition, they were also asked whether they think it is important for their health provider to know about their sexual orientation and gender identity, and whether they would be willing to answer these questions on a registration form.

Prior to survey administration, study staff piloted the survey with eight staff from The Fenway Institute and a staff member at the Center for American Progress, who provided comments, suggestions, and noted the time it took to complete the survey. The study team discussed these suggestions and incorporated them into the survey.

### Participant Recruitment and Survey Administration

Each site developed its own recruitment and implementation plan to enroll participants there within a two-week period. Three of the four sites had a dedicated staff person to administer the survey. Study staff approached potential participants in the clinic waiting room or at the registration desk, asked if they were interested in completing a short survey, and provided an information sheet outlining key elements of the study. If interested, the participant completed the survey and received a $10 gift card. If participants were called into the medical visit before completing the survey, they completed the remainder of the survey after the visit.

## Results

In 2013, 301 participants were surveyed about their experience with answering SOGI questions in clinical settings at four community health centers, including Fenway Health, Howard Brown, Chase Brexton, and Beaufort. A total of nine potential participants refused to complete the survey at the four sites, citing lack of interest or time limitations. Fifty-one percent of respondents identified as “straight or heterosexual.” Most respondents from the Beaufort Health Center network in rural South Carolina (78%) said they were straight or heterosexual, as did 45% of respondents at Chase Brexton in Baltimore, 34% of respondents at Fenway Health in Boston, and 36% of respondents at Howard Brown in Chicago. Twenty-five percent of respondents from the 4 locations said they were gay, lesbian, or homosexual, with a range from 5% gay/lesbian/homosexual at Beaufort in South Carolina to 42% at Howard Brown in Chicago. An average of 7% self-identified as bisexual—ranging from 0% at Beaufort to 15% at Howard Brown (Table 1).

**Table 1:** Demographic characteristics of participants surveyed and their association with the likelihood of answering that it is important to ask patients about sexual orientation and gender identity when they register at the health center.

		Q7. Sexual Orientation		Q8. Gender Identity	
	N = 301 (% of total sample)	*χ* ^2^	p-value	*χ* ^2^	p-value
**Race**		4.216	0.040*	4.949	0.084
Black/African American	123 (41%)				
Asian**	3 (1%)				
Caucasian	132 (44%)				
Multiracial	15 (5%)				
Native American/Alaskan Native/Inuit**	6 (2%)				
Pacific Islander**	2 (1%)				
Other**	16 (5%)				
Missing answer	4 (1%)				
**Ethnicity**		1.901	0.168	0.257	0.612
Hispanic/Latino/Latina	24 (8%)				
Not Hispanic/Latino/Latina	234 (78%)				
Missing answer	43 (14%)				
**Sexual Orientation**		7.337	0.007*	2.775	0.096
Lesbian, gay or homosexual	76 (25%)				
Straight or heterosexual	154 (51%)				
Bisexual**	22 (7%)				
Something else**	29 (10%)				
Don't know**	16 (5%)				
Missing answer	4 (1%)				
**Age Group**		3.588	0.310	9.967	0.019*
18–29 years old	89 (29.6%)				
30–49 years old	112 (37.2%)				
50–64 years old	79 (26.2%)				
65 or older	21 (7.0%)				
**Health Center**		0.380	0.944	1.490	0.685
Beaufort	100 (33%)				
Chase Brexton	67 (22%)				
Fenway	101 (34%)				
Howard Brown	33 (11%)				

* Statistically significant at the level of p<0.05

**Category was not included in the chi-square analysis due to small sample size

Q7. As part of a written registration form, do you think it is important to ask patients about sexual orientation when they register at the health center?

Q8. As part of a written registration form, do you think it is important to ask patients about gender identity when they register at the health center?

Note: Data may not add up to 100% due to rounding.

Forty-seven of 301 respondents were transgender. Some 5.3% percent of respondents identified as Male-to-Female (MTF)/Transgender Female/Trans Woman; 10.3% identified as Female-to-Male (FTM)/Transgender Male/Trans Man. Together they were 15.6% of our sample. The range across sites was from 1 transgender patient out of 100 (1%) at Beaufort to 20 of 67 (29.8%) patients at Chase Brexton.

The sample was racially diverse: 44% White, 41% Black, 5% other, 5% multiracial, 2% Native American/Alaskan Native, and 2% Asian or Pacific Islander. Eight percent were Hispanic/Latino/Latina (Table 1). Thirty percent were age 18–29, 37% age 30–49, 26% age 50–64, and 7% age 65 or older.

Nearly 3 in 4 respondents from the 4 CHCs said that asking about sexual orientation on registration forms is important (74% agreed that this was important versus 25% who disagreed) (Table 2). An even greater majority agreed that asking about gender identity is important (82% versus 17%).

**Table 2:** Responses to survey questions 10, 15, 16, and 17.

	1 - Strongly Disagree	2 - Somewhat Disagree	3 – Neutral	4 - Somewhat Agree	5 – Strongly Agree	Missing answer	Mean (SD)
N = 301	n (%)	n (%)	n (%)	n (%)	n (%)	n (%)	n (%)
*10. In answering the question about sexual orientation, please tell us whether you agree or disagree:*							
10a. I understood what the question was asking about me	21 (7.0%)	5 (1.7%)	18 (6.0%)	27 (9.0%)	225 (74.8%)	5 (1.7%)	4.45 (1.15)
10b. I understood all of the answer choices	19 (6.3%)	3 (1.0%)	9 (3.0%)	32 (10.6%)	228 (75.7%)	10 (3.3%)	4.54 (1.08)
10c. The question was easy for me to answer	20 (6.6%)	5 (1.7%)	11 (3.7%)	32 (10.6%)	225 (74.8%)	8 (2.7%)	4.49 (1.12)
10d. I would answer this question on a registration form at this health center.	22 (7.3%)	3 (1.0%)	16 (5.3%)	33 (11.0%)	217 (72.1%)	10 (3.3%)	4.44 (1.15)
10e. This question allows me to accurately document my sexual orientation	26 (8.6%)	8 (2.7%)	23 (7.6%)	39 (13.0%)	195 (64.8%)	10 (3.3%)	4.27 (1.26)
10f. I think this information is important for my medical provider to know about me	24 (8.0%)	10 (3.3%)	25 (8.3%)	37 (12.3%)	197 (65.4%)	8 (2.7%)	4.27 (1.25)
*15. In answering Question 13 (“What is your current gender identity?”), please let us know whether you agree or disagree:*							
15a. I understood what the question was asking about me	20 (6.6%)	3 (1.0%)	7 (2.3%)	20 (6.6%)	246 (81.7%)	5 (1.7%)	4.58 (1.08)
15b. I understood all of the answer choices	20 (6.6%)	9 (3.0%)	8 (2.7%)	25 (8.3%)	234 (77.7%)	5 (1.7%)	4.50 (1.14)
15c. The question was easy for me to answer	20 (6.6%)	1 (0.3%)	9 (3.0%)	21 (7.0%)	244 (81.1%)	6 (2.0%)	4.59 (1.07)
15d. I would answer this question on a registration form at this health center.	21 (7.0%0	3 (1.0%)	14 (4.7%)	17 (5.6%)	242 (80.4%)	4 (1.3%)	4.54 (1.12)
*16. In answering Question 14 (“what sex were you assigned at birth on your original birth certificate?”), please let us know whether you agree or disagree:*							
16a. I understood what the question was asking about me	17 (5.6%)	1 (0.3%)	8 (2.7%)	11 (3.7%)	256 (85.0%)	8 (2.7%)	4.67 (1.00)
16b. The question was easy for me to answer	19 (6.3%)	1 (0.3%)	12 (4.0%)	11 (3.7%)	247 (82.1)	11 (3.7%)	4.61 (1.06)
16c. I would answer this question on a registration form at this health center.	20 (6.6%)	4 (1.3%)	14 (4.7%)	15 (5.0%)	238 (79.1%)	10 (3.3%)	4.54 (1.12)
*17. In answering the gender identity questions (which includes questions 13 and 14), please let us know whether you agree or disagree:*							
17a. This set of questions allows me to accurately document my gender identity	20 (6.6%)	5 (1.7%)	17 (5.6%)	18 (6.0%)	231 (76.7%)	10 (3.3%)	4.49 (1.14)
17b. I think this information is important for my provider to know about me	20 (6.6%)	7 (2.3%)	14 (4.7%)	22 (7.3%)	227 (75.4%)	11 (3.7%)	4.48 (1.15)

Note: Data may not add up to 100% due to rounding.

Most respondents agreed that they “understood what the question was asking about me.”

The average response of LGB respondents was significantly greater (t = 3.326, p = 0.001). However, the means of both groups were in the agreement range (mean response: 4.80 (SD = 0.690) vs. 4.40 (SD = 1.22).

Most respondents agreed that “The question was easy for me to answer.” The difference between LGB respondents and heterosexual respondents (mean response: 4.73 (SD = 0.778) vs. 4.49 (SD = 1.16)) was not statistically significant (t = 1.885, p = 0.061).

Most respondents agreed that “I would answer this question on a registration form at this health center.” The average response of LGB respondents was significantly greater (t = 2.806, p = 0.005). However, the means of both groups were in the agreement range (mean response: 4.73 (SD = 0.736) vs. 4.38 (SD = 1.22).

Most respondents agreed that “This question allows me to accurately document my sexual orientation.” The average response of LGB respondents was significantly greater (t = 2.156, p = 0.032). However, the means of both groups were in the agreement range (mean response: 4.55 (SD = 0.92) vs. 4.24 (SD = 1.37).

Seventy-eight percent of all respondents somewhat agreed or strongly agreed that sexual orientation “information is important for my medical provider to know about me.” Survey respondents were invited to write comments about the sexual orientation and gender identity questions. One respondent wrote, “I think my relationship with my doctor should reflect my sexual orientation because it provides better care.” Only 11% of those surveyed disagreed somewhat or strongly that sexual orientation information was important for their provider to know about them, while 8% were neutral (Table 2).

When asked if they would make changes to the sexual orientation question, 15% said they would ask the question differently, while 83% said that they would not make changes to the sexual orientation question tested.

Most respondents were able to answer the two-part gender identity question. Only 1% (n = 3) declined to answer the current gender identity question. Only 0.3% chose “genderqueer” (n = 1) and only 0.3% chose “other” (n = 1); the other 98% chose from among the gender identity options. Only two percent declined to answer the question, “What sex were you assigned on your original birth certificate?”

Seventy-eight percent of all respondents strongly agreed that they understood all the choices in the gender identity question, while only 7% strongly disagreed. Heterosexual respondents were more likely than LGB respondents to say they did not understand all the choices of responses in the gender identity question. Eighty-four percent of all respondents strongly or somewhat agreed that they would answer the birth sex question, and 86% strongly or somewhat agreed that they would answer the current gender identity question. Most transgender respondents agreed that the gender identity question allowed them to accurately document their gender identity. Nine in ten of all respondents (90%) said they would not change the gender identity questions, while 7% would. Eighty-eight percent of male and female non-transgender respondents somewhat or strongly agreed that they would answer the gender identity question on a registration form at their health center.

Several transgender respondents raised concerns about being asked their sex assigned at birth. One wrote, “Though I understand the importance of knowing birth sex when dealing with trans medical issues, it's still a very sensitive question that most [transgender people] would probably not want to answer.” Overwhelming majorities of all groups—transgender and non-transgender men and women—strongly agreed that “this information is important for my provider to know about me.”

Some respondents said that they wanted their providers to ask them about their sexual orientation and gender identity; while they agreed that it should be in their medical record, they questioned whether it should be asked at registration. A few expressed concerns about privacy of data, and a few commented on the importance of training staff on why SOGI data are being gathered and why knowing a patient's sexual orientation and gender identity is important for providing culturally competent and affirming care and understanding LGBT health disparities.

There were no significant differences among the average responses to SOGI questions of the 7 racial groups when using an ANOVA test.

There were also statistically significant differences between those 65 and older and those younger than 65. Older participants tended to provide lower rankings for the following questions:

I understood what the [sexual orientation] question was asking about me. (t = 7.959, p = 0.010)

I understood all of the [sexual orientation] answer choices. (t = 6.929, p = 0.015)

I understood what the [gender identity] question was asking about me. (t = 4.695, p = 0.041)

I understood all of the answer choices [gender identity question]. (t = 4.836, p = 0.039)

Over 65 responses to these questions were in the 3, or neutral, range on the Likert scale. However, on 11 other questions about the SOGI question, including whether they would answer the questions on a registration form, there were no significant age differences between elders and middle age and younger respondents.

## Discussion

This evaluation of questions about sexual orientation and gender identity among a diverse group of patients at four CHCs shows widespread understanding of these questions and willingness to answer them, both among LGBT respondents and among heterosexual and non-transgender respondents. Most LGB respondents said that the sexual orientation question accurately reflected their identities and that they would not change the wording of the questions. They also understood why it is important for providers to know about their sexual orientation. This indicates broad support among LGB patients, as well as among heterosexual patients, for sexual orientation data collection in clinical settings. These findings also correlate with findings from a recent nationwide study of more than 860 LGBT individuals with incomes under 400% of the poverty level, in which 76% of respondents said it is important to be open with their providers about their SOGI and 74% indicated that they are “out” to their provider about their SOGI. [24]

The two-step gender identity question (current gender identity and birth sex) was also widely understood by all patients surveyed. It is worth noting that majorities believed that it was important for providers to know about their patients' gender identity, and would be willing to answer the question in their care setting. Further research, including focus groups, would be helpful regarding concerns among some transgender respondents with regard to answering the sex assigned at birth question. It is important to note that most transgender respondents indicated that they would answer both parts of the gender identity question—current gender identity and sex assigned at birth.

A two-step gender identity question is becoming more widely adopted in health data systems. In addition to its endorsement by leading transgender researchers in the U.S. (Center of Excellence for Transgender Health, GENIUSS) [21] and globally (WPATH), [22] the Centers for Disease Control and Prevention (CDC) adopted the two-step gender identity and birth sex question for use in their Adult Case Report Form and in their electronic surveillance system, the Enhanced HIV/AIDS Reporting System. [25] In a recent analysis, the two-step question was found to have near-zero missing data and to result in a transgender-spectrum response rate twice that elicited by a single question that asked respondents to select from four response options for their sex (male, female, transgender, other). [26]

Membership in several demographic groups was not associated with being more likely to think that it is important to ask about sexual orientation on registration forms (Table 1). Respondents were equally likely answer “yes,” regardless of ethnicity (*χ* ^2^ = 1.901, *P* = 0.168), age group (*χ* ^2^ = 3.588, *P* = 0.310), gender identity (*χ* ^2^ = 2.132, *P* = 0.344), and health center location (*χ* ^2^ = 0.380, *P* = 0.944). Respondents who identified as lesbian, gay, or homosexual were more likely than the straight or heterosexual group to think it was important (*χ* ^2^ = 7.337, *P* = 0.007). Our sample wasn't large enough to analyze many of the race categories but we found that Black/African Americans were more likely to answer “yes” to this question than Caucasians (*χ* ^2^ = 4.216, *P* = 0.040).

There were no significant group differences for race (*χ* ^2^ = 4.949, *P* = 0.084), ethnicity (*χ* ^2^ = 0.257, *P* = 0.612), sexual orientation (*χ* ^2^ = 2.775, *P* = 0.096), or health center location (*χ* ^2^ = 1.490, *P* = 0.685) in their likelihood to think that it is important to ask about gender identity on registration forms. The exception was that the two older groups answered “yes” less often than expected the two younger groups answered “yes” more often (*χ* ^2^ = 9.967, *P* = 0.019).

Respondents overwhelmingly expressed support for asking SOGI questions and understood the importance of providers' knowing their patients' SOGI. While there were some significant differences between elders and other respondents, there were no statistically significant age differences in terms of willingness to answer SOGI questions on a registration form and understanding the importance of providers' knowing this information about their patients.

The Centers for Medicare and Medicaid Services (CMS) and the Office of the National Coordinator of Health Information Technology (ONC) are currently considering whether to include SOGI data collection in the Stage 3 guidelines for the incentive program promoting meaningful use of electronic health records. During the Stage 2 Meaningful Use Guidelines process, the federal government made the following pronouncement:

Considering the lack of consensus for the definition of the concept of gender identity and/or sexual orientation as well as for a standard measure of the concept and where it would be most appropriate to store the data within the EHR, we will await further development of a consensus for the goal and standard of measurement for gender identity and/or sexual orientation…[27]

We believe that this study demonstrates that a consensus exists regarding the importance of sexual orientation and gender identity information for the provision of optimal clinical care, and that the measures developed in this study could function as standard measures that could be employed in real-world health care settings. The SOGI questions tested in these four settings could, if widely used, be acceptable to patients across the country—LGBT and straight, Black and White, older and younger—and could provide important information on patients that can help us better understand health disparities affecting LGBT people.

As many speakers at the October 2012 Institute of Medicine workshop on LGBT data collection in EHR systems noted, buy-in from staff, including front desk staff as well as providers, is essential to effective SOGI data collection. Furthermore, SOGI data collection should be coupled with cultural competency training in which staff can ask questions and work through any discomfort or misunderstandings they may have. Such training should occur in the context of training health professionals and administrative staff about broader issues of achieving quality care with diverse patient populations. [20]

There are limitations to consider when interpreting findings. First, we surveyed a sample of each clinic population regarding SOGI questions. If this sample differed from the actual patient population, then this may have biased our results. However, there is no reason to believe, given the high rates of participation, that the samples surveyed differed from the general patient populations of each health clinic. Second, each clinic surveyed patients using different methodologies during a two week period. Because the survey collection occurred over a brief period, not all health center patients had the opportunity to complete a survey. Third, each site only surveyed patients who arrived for appointments. Any patient who did not keep his or her appointment on a particular survey day did not have an opportunity to complete the survey. Fourth, since the surveys were administered in busy clinics, we did not want to interfere with clinic workflow, so the survey length was limited.

The primary strength of this study was the regional, racial, and age diversity of the patients who responded to the survey. Since we are concerned with asking SOGI questions in a clinical environment, we conducted the study with patients in both urban and rural areas in four different community health centers in different regions of the U.S. Therefore, we were able to reach patients with different backgrounds, including racially diverse backgrounds, who may have different opinions on the importance of SOGI data. An additional strength is that not all of the health centers where we tested these SOGI questions were LGBT-focused. Including a clinic that was not LGBT-focused and located in a rural community strengthened the generalizability of the results. A full range of ages and educational levels were represented among the survey respondents. Additionally, the survey provided patients with an opportunity to comment on these questions so that any new or unanticipated issues could be expressed. These results provide evidence suggesting that asking SOGI questions in clinical settings is both feasible and important for facilitating communication between patients and clinicians.

## Conclusion

This survey of a diverse group of patients in four health centers finds that most patients understand the importance of asking about sexual orientation and gender identity and would be willing to answer a set of existing questions developed to collect SOGI data in health care settings. We believe that health care providers and regulatory bodies should move forward by taking steps to facilitate SOGI data collection in clinical settings and in EHRs. In particular, inclusion of SOGI questions in the standard demographic section of the Stage 3 meaningful use guidelines is an important step that the Centers for Medicare and Medicaid Services and the Office of the National Coordinator for Health Information Technology can take to advance SOGI data collection. This would be consistent with our findings of widespread agreement among survey respondents regarding the acceptability of SOGI questions, as well as with the emphasis placed on SOGI data collection and LGBT health in recent years by entities such as the Institute of Medicine, The Joint Commission, and the Department of Health and Human Services itself.

## Supporting Information

Appendix S1 (DOCX)
