# Supplementary material for: Do Ask, Do Tell: High Levels of Acceptability by Patients of Routine Collection of Sexual Orientation and Gender Identity Data in Four Diverse American Community Health Centers
Source: PLoS One. 2014 Sep 8;9(9):e107104. doi: 10.1371/journal.pone.0107104 (PMC4157837; doi:10.1371/journal.pone.0107104)
Supplement: Appendix S1 — (DOCX) [file pone.0107104.s001.docx]

**Appendix S1**

Data Analysis

The survey data from each site was entered into a password-protected REDCap database.  Data from all sites was exported from REDCap to SPSS version 18 for analysis. All analysis was performed and tables were created in SPSS. Mean responses to survey questions using the Likert scale were compared with ANOVA and t-tests. In cases where Levene’s test for the equality of variances was significant, equal variances of group responses were not assumed and t-test results were reported accordingly.  Univariate comparisons were performed using Pearson’s chi-squared test for categorical variables. Statistical significance was determined by a p-value < 0.05.

In 13 cases, multiple gender identity responses were re-coded into a “primary gender identity” variable for the purposes of data analysis. The transgender categories took first precedence in the re-coding of multiple gender identity responses: Female-to-Male (FTM)/Transgender Male/ Trans Man and Male-to-Female (MTF)/Transgender Female/Trans Woman. If transgender responses were not chosen, then male or female were second, then “Genderqueer,” and “Other” was last. The answer to the birth sex question was used to confirm re-coded responses for gender identity.

IRB

The IRBs at The Fenway Institute (covering Fenway, Chase-Brexton and Beaufort-Jasper- Hampton) and Howard Brown approved a waiver of documented informed consent due to the anonymous nature of the study and the absence of any personal identifiers attached to the survey. However, participants were provided with a “research information form” for participation in this study (included with informed consent waiver). This form was handed out with the paper survey and included all the essential elements of an informed consent. Continuing on to answer the survey questions indicated that participants voluntarily agreed to participate.

Survey Recruitment at the Four Sites

At the 4 sites, all consecutive patients were approached during the time period in which patients were being asked to complete the survey. There were no other inclusion or exclusion criteria besides those already stated.

*Fenway:* Fenway Institute staff recruited from the registration desk on each of three medical floors in the Fenway Health main building at 1340 Boylston St., Boston. Only two potential respondents declined to complete the survey, citing lack of time.

*Beaufort:* The original implementation plan for Beaufort consisted of staff affiliates distributing the questionnaire to all patients at the time of registration and prior to the patient seeing the provider. If the waiting time was brief, the patient was permitted to take the survey into the exam room for completion. The finalized questionnaires were to be returned to the medical office assistant in order to receive the gift card; at the end of the day, the research assistant would be accountable for assembling the completed questionnaires from the registration staff. However, in an effort to get the questionnaires completed in an efficient and expeditious manner, minor modifications were made to the implementation plan.

The surveys were implemented at two individual sites, Hardeeville Medical Center (Adult Medicine) and Port Royal Medical Center (Adult Medicine, WIC, and OB/GYN), which is one of Beaufort’s busiest sites. The research associate ascertained patients’ interest in participating in the survey, administered the survey while the patients were in the waiting area, and presented the gift card after completion. Only two individuals declined to participate in the survey, and their reason for non-participation was due to the fact that they did not receive their care at Beaufort.

*Chase Brexton:* Participants were recruited at two sites: Mount Vernon in downtown Baltimore and Columbia Center in Columbia, MD. Chase Brexton experienced challenges to study recruitment, including the loss of a research assistant and a move from one building to another for their largest clinical site. As a result, they had difficulty reaching their original recruitment goal and revised their recruitment plan. At the Columbia, Maryland site, a research assistant recruited participants while they waited for their provider visit. At the downtown Baltimore site, several providers, social workers, and a preventive health outreach worker recruited participants directly during their visits.

*Howard Brown:*  Howard Brown Health Center (HBHC) staff first attended the agency’s transgender female support group. Here information was provided and members were asked to consider completing the questionnaire. A few days later, staff spent an afternoon in the HBHC clinic waiting area, where the study was introduced, patients read the study information page, and completed the questionnaire.  In both instances the participants were given the information page and questionnaire in closed envelopes which they sealed upon completion. The sealed envelopes were returned to the HBHC Site Study Coordinator.
